# Repurposing Neurological Drugs Against Dengue Virus Infection

**DOI:** 10.1155/jotm/8376728

**Published:** 2025-10-04

**Authors:** Ming-Kai Jhan, Ting-Jing Shen, Thi Thuy Nguyen, Chiou-Feng Lin

**Affiliations:** ^1^Department of Microbiology and Immunology, School of Medicine, College of Medicine, Taipei Medical University, Taipei City, Taiwan; ^2^School of Medical Laboratory Science and Biotechnology, College of Medical Science and Technology, Taipei Medical University, New Taipei City, Taiwan; ^3^Department of Oncology, Hue University of Medicine and Pharmacy, Hue University, Hue City, Vietnam; ^4^Graduate Institute of Medical Sciences, College of Medicine, Taipei Medical University, Taipei City, Taiwan

**Keywords:** CNS, dengue fever, dengue virus, drug repurposing, nervous system–acting compounds, nervous system–targeted compounds, neurological complications of dengue, potential treatment options

## Abstract

Dengue fever is highly prevalent in tropical and subtropical regions, where it is caused by dengue virus (DENV) and transmitted by arthropods. While DENV infection manifests with a spectrum of clinical symptoms, severe cases can lead to hemorrhagic fever and shock syndrome. Increasing evidence over the past decade has highlighted the neurological complications associated with DENV; however, the underlying mechanisms remain poorly understood. Despite this knowledge gap, various central nervous system (CNS)–targeted drugs have shown promise in mitigating DENV-induced neurological impairment. In this review, we provide an overview of the neurotropic features of DENV and summarize current evidence on the antiviral effects of selected nervous system-acting compounds. We also explore the potential mechanisms by which these agents may reduce DENV infection. Given the urgent need for effective dengue therapeutics, repurposing CNS-targeting drugs represents a promising strategy. Finally, we examine the potential and underlying mechanisms for employing neuropharmaceutical agents as one of the antiviral therapies for DENV infection.

## 1. Introduction

Dengue fever (DF) is an arthropod-borne viral disease that poses a major public health issue in tropical and subtropical regions, causing thousands of infections annually. The global incidence of DF has increased significantly due to causes such as urbanization and climate change. These factors promote the expansion and proliferation of mosquitoes such as *Aedes aegypti* and *Aedes albopictus*, which are the primary vectors of the disease. DF is caused by the dengue virus (DENV), a positive single-stranded RNA virus belonging to the *Flaviviridae* family. Upon DENV infection, diagnosis can be established using RT-PCR or NS1 antigen detection during the acute phase, while serological testing (IgM/IgG) is useful in later stages to confirm the infection. Clinically, DF progresses through three distinct phases: febrile, critical, and convalescent. The febrile phase typically manifests 7–10 days postinfection and is characterized by high fever, retro-orbital pain, myalgia, arthralgia, nausea, vomiting, lymphadenopathy, and rash. Approximately 80% of cases recover without complications; however, in about 20% of patients, the disease progresses to a critical phase, typically occurring between Days 3 and 7 around the time of defervescence [1–3]. This critical phase is marked by an increased vascular permeability and warning signs such as severe abdominal pain, hepatomegaly, pleural effusion, mucosal bleeding, and hematological abnormalities, including alterations in hematocrit and platelet count. Hemorrhagic manifestations such as petechiae, epistaxis, hematemesis, melena, and hematuria may also be observed, potentially leading to severe forms of the disease, including dengue hemorrhagic fever (DHF) and dengue shock syndrome (DSS) [4, 5]. There is currently no specific medication for DENV. Supportive care is essential for preventing DHF and DSS, primarily through fluid resuscitation and hemodynamic stabilization [5]. Notably, approximately 95% of affected individuals recover within 24–48 h with appropriate medical management [6, 7]. However, recent evidence suggests that DENV may exhibit neurotropic potential. This review explores DENV-related neurological complications and examines the potential of repurposed neuroactive drugs as antiviral agents. We first outline the clinical features and possible mechanisms of DENV neuroinvasion. Next, we summarize current evidence on neuroactive compounds with antiflaviviral potential, organized by their mechanisms of action. Finally, we discuss knowledge gaps, safety considerations, and future research directions.

## 2. Neurological Complications Caused by DENV

Neurological complications associated with DENV infection have been increasingly documented over the past decade [8–21]. Historically, neurological complications were considered rare in DF patients because DENV was initially thought to be non-neurotropic, compared with other well-established neurotropic flaviviruses such as Japanese encephalitis virus (JEV), Zika virus (ZIKV), and West Nile virus (WNV). Notably, DF and Japanese encephalitis (JE) can be clinically distinguished by the primary manifestations. DF typically presents with high fever, myalgia, rash, and thrombocytopenia. In contrast, JE primarily presents with acute encephalitis, altered mental status, and seizures [22]. However, the detection of DENV antigens in postmortem brain tissues, along with the presence of dengue-specific IgM antibodies in cerebrospinal fluid (CSF), provides clinical evidence supporting the neurotropic potential of DENV [9, 23].

In recognition of these findings, the World Health Organization (WHO) revised its dengue classification in 2009 (Table 1). Central nervous system (CNS) involvement was designated as a severe manifestation of dengue disease [3]. Since then, reports of neurological complications associated with severe dengue have continued to rise [24–27]. WHO guidelines and literature indicate that DENV-related neurological manifestations can be categorized into encephalitis, encephalopathy, neuromuscular disorders, and neuro-ophthalmic conditions [3, 24]. Among hospitalized dengue patients, neurological complications—including encephalitis and encephalopathy—have been reported in approximately 0.5%–7.4% of cases [24]. Despite increasing recognition of these complications, the precise mechanisms by which DENV invades and affects neuronal tissues remain poorly understood. Further research is required to elucidate the pathophysiological pathways underlying DENV-associated neuroinvasion and its clinical implications.

Currently, the management of neurological complications in dengue patients remains largely supportive, as no licensed prophylactic or therapeutic agents specifically targeting DENV infection are available [3]. Dengvaxia, the first antidengue vaccine approved by the U.S. Food and Drug Administration (FDA), has been licensed in several dengue-endemic countries. However, its use has been restricted due to safety concerns, particularly the increased risk of severe dengue in individuals without prior DENV exposure. In addition to its limited duration of cross-protective efficacy, Dengvaxia has also been associated with an elevated risk of severe disease upon secondary infection with heterologous DENV serotypes [28].

Additionally, another antidengue vaccine, Qdenga, was first licensed by the European Union in 2022 [29]. Qdenga is constructed on an attenuated DENV2 backbone that coexpresses the structural proteins of DENV1, DENV3, and DENV4. In contrast, Dengvaxia is a live attenuated chimeric vaccine constructed on a yellow fever virus (YFV) backbone. Notably, Qdenga has demonstrated protective efficacy in both seropositive and seronegative individuals. However, several concerns remain regarding Qdenga's long-term efficacy, its limited serotype-specific protection particularly against DENV3 and DENV4 in seronegative individuals, and also, the vaccine safety usage in adults over 60 years of age remains unclear [30].

To date, no specific antiviral therapies have been approved for the treatment of DENV infection, particularly in cases involving CNS complications. This review explores the therapeutic potential of repurposing clinically approved neuroactive drugs as antiviral agents against DENV, with an emphasis on their mechanisms of action and relevance to neurotropic manifestations of the disease.

## 3. DENV Structure, Life Cycle, and Neuroinvasion Potential

In virology, DENV infection is believed to initiate in skin-resident cells such as keratinocytes at the site of the arthropod bite. The virus subsequently spreads through the bloodstream and infects immune cells, which serve as major sites for viral replication. DENV infection further triggers immune activation and cytokine release, and the resulting overactive immune response may lead to vascular leakage and systemic inflammation [31].

DENV exists as four distinct serotypes: DENV-1, DENV-2, DENV-3, and DENV-4. The mature DENV particle is approximately 50 nm in size with a spherical structure composed of lipid envelope (E), membrane (M), and capsid (C) proteins. The virion contains a single-stranded, positive-sense RNA genome of approximately 11 kb in length, which encodes three structural proteins (C, precursor membrane protein [prM], and E) and seven nonstructural proteins (NS1, NS2A, NS2B, NS3, NS4A, NS4B, and NS5). The structural proteins form the outer layer of the virion and responsible for viral entry and assembly, whereas the nonstructural proteins play a critical role in viral replication, assembly, and the inhibition of host immune responses [32, 33].

DENV binds to host cells through interactions between the viral envelope (E) protein and various cellular receptors. Identified receptors involved in this process include heparan sulfate (HS), heat shock proteins (HSP90 and HSP70) [34, 35], glucose-regulated protein 78 (Grp78/BiP) [36], CD14 [37], DC-SIGN [38], CD206 [39], and CLEC5A [40]. In brief, DENV enters host cells via clathrin-mediated endocytosis. Acidification within endosomes induces conformational changes in the E protein, facilitating nucleocapsid release into the cytoplasm. The viral RNA is released into the cytoplasm and translated at the endoplasmic reticulum (ER) to produce a single polyprotein, which is cleaved by host and viral proteases into structural and nonstructural proteins. Viral replication occurs through a negative-strand RNA intermediate, and newly synthesized genomes are packaged and assembled with structural proteins in the ER. Immature virions undergo maturation in the trans-Golgi network via furin-mediated prM cleavage, leading to E protein rearrangement. Mature virions are then released by exocytosis to infect new cells [41–43].

DENV targets a variety of cells such as dendritic cells, monocytes, and B cells. Viral entry and replication subsequently activate monocytes, macrophages, and dendritic cells, initiating a cascade of cellular immune responses including the production of proinflammatory cytokines and chemokines. Excessive release of these immune mediators may lead to cytokine storm, resulting in plasma leakage and multiorgan failure. However, the precise mechanisms by which DENV gains access to the CNS remain unclear. The most widely proposed route of CNS invasion is via the bloodstream. Some studies suggest that DENV may cross the blood–brain barrier (BBB) either through infected cells (the “Trojan horse” mechanism) or by directly infecting endothelial cells and disrupting tight junction integrity [44–46].

In the CNS, neurons account for approximately 10%–20% of brain cells, while astrocytes comprise 20%–40%, and microglia around 5%–15% [47], although DENV has been detected in astrocytes in postmortem brain tissues [48]. However, the role of astrocytes in DENV neuropathogenesis remains controversial. In murine models, intraperitoneal injection of neuro-adapted DENV strains did not detect viral protein expression in astrocytes [49, 50]. This suggests that astrocytes may not be directly susceptible. Further studies are needed to determine whether they are direct targets or contribute through neuroinflammatory responses.

Meanwhile, several studies have indicated that microglia—the resident immune cells of the brain—are among the target cells for DENV infection in the CNS. Studies using the murine microglial cell line BV2 showed that these cells are susceptible to all four DENV serotypes [51]. Meanwhile, other studies demonstrated that viral entry occurs primarily through clathrin-mediated endocytosis [52]. In a neonatal murine model of DENV encephalitis, microglia were found to express the DENV envelope (E) protein following infection. Furthermore, microglial depletion in this model reduced peripheral immune cell infiltration and inflammatory cytokine secretion, partially alleviating neuronal damage in the CNS [53]. These findings suggest that microglial activation is one of the key contributors to DENV-induced neurotoxicity.

In addition to glial cells, neurons are also potential targets of DENV infection in the CNS.

Neuronal damage may result not only from immune activation but also from direct infection by the virus. Studies have demonstrated that DENV can directly infect neurons both in vivo and in vitro. The murine cell line Neuron-2a produces mature virions after DENV infection [54]. In vivo murine models also show that neurons can be directly infected by DENV [55].

Taken together, increasing evidence suggests that DENV possesses neuroinvasive potential. Many neuroactive drugs are capable of crossing BBB. Their pharmacological properties may offer therapeutic advantages in mitigating DENV-associated neuroinflammation.

## 4. Current Developments and Targets of Antidengue Agents

Although no specific licensed anti-DENV medication is currently available, numerous research are dedicated to discovering and developing potential therapeutic agents. DENV infection inevitably involves interactions with host factors and intracellular pathways throughout the viral life cycle. As such, these therapeutic agents can be categorized into two groups based on their mechanisms of action.

### 4.1. Direct-Acting Antivirals (DAAs)

DAAs are small molecules or compounds designed to specifically target interactions between viral proteins or genomes, thereby minimizing off-target effects on host cells due to their specificity [56]. The viral E protein is the primary target for DAA development, owing to its crucial role in viral–host interactions. The C-terminal portion of the DENV E protein is involved in the viral fusion process, making it an attractive target site for DAAs. A synthetic DV2 peptide derived from the C-terminal region of the DENV E protein was shown to inhibit all four DENV serotypes [57]. It also reduced ZIKV infection in Vero cells and improved survival in ZIKV-infected mice [58].

In 2019, scientists discovered a cyanohydrazone compound (3-110-22) and its analogs that exhibit inhibitory activity against several flaviviruses, including JEV, ZIKV, and DENV. A derivative, JBJ-01-162-04, was shown to block a DENV E protein-mediated membrane fusion in vitro and significantly reduced serum viremia in a dose-dependent manner in DENV-infected AG129 mice [59]. Also, the DENV NS2B-NS3 protease complex is a potential target for DAA development. A cell-based study showed that the small molecule SK-12 inhibited all four DENV serotypes by disrupting NS2B-NS3 interaction and reducing protease activity [60].

High-throughput screening identified several potential antivirals targeting the DENV NS2B-NS3 complex, including temoporfin, niclosamide, and nitazoxanide (NTZ). Temoporfin showed the strongest inhibition of virion production and, along with niclosamide, was found to bind key residues on NS3, blocking protease activity [61]. Niclosamide, an antiparasitic drug, also reduced DENV-induced mortality and encephalitis-like symptoms in mice and inhibited endosomal acidification to block viral fusion and RNA release [62]. Other studies suggest it may also impair virion maturation through multiple host-directed mechanisms [63].

NTZ, an antiparasitic prodrug, inhibits the protease activity of the DENV NS2B-NS3 complex. Its active metabolite, tizoxanide (TIZ), reduces DENV virion production in a dose-dependent manner. While TIZ does not block viral entry, it suppresses replication and may interfere with multiple stages of the viral life cycle, including RNA accumulation, virion release, and production of noninfectious particles [64].

Most studies on DAAs have been limited to cell-based experiments, with only a few evaluated in immunodeficient murine models. Consequently, important pharmacological properties such as half-life, cellular permeability, and in vivo safety remain to be fully characterized. Furthermore, the high genetic variability of RNA viruses compared to DNA viruses presents additional challenges for the development of effective DAAs.

### 4.2. Host-Directed Antivirals (HDAs)

In contrast to DAAs, HDAs target host factors to disrupt the interactions between the virus and its host. Their mechanisms of action may include regulation of host signaling pathways, modulation of host proteins, and enhancement of antiviral immunity. Neurological complications have been reported in DENV infections, with a range of clinical manifestations. These include headaches, blurry vision, ocular pain, altered consciousness, irritability, and insomnia. In rarer instances, more severe neurological symptoms such as seizures, focal neurological deficits, encephalitis, encephalopathy, and neuro-ophthalmic manifestations have been observed. Several widely used nervous system-acting compounds have been reconsidered for their potential antidengue effects attributed to their HDA functions.

Prochlorperazine (PCZ), a first-generation antipsychotic commonly used to treat psychosis, nausea, vomiting, and headaches, has demonstrated antiviral properties against DENV. PCZ prevents DENV binding and entry by targeting the dopamine D2 receptor (D2R) and inhibiting clathrin-associated endocytosis. In an immunodeficient murine model, PCZ treatment—via multiple routes—has been shown to delay DENV-induced lethality, either fully or partially [65]. Further studies revealed that JEV also exploits D2R for cellular infection. PCZ treatment was found to inhibit JEV infection in human neuronal cells [66]. Similarly, chlorpromazine (CPZ), a phenothiazine with a structure similar to PCZ, has been shown to inhibit DENV entry in murine microglial cell lines. Pretreatment with CPZ disrupts clathrin-mediated endocytosis, a primary pathway for DENV entry into host cells [52].

A recent study by an Argentine research group showed that trifluoperazine (TFP), another antipsychotic used to treat schizophrenia and anxiety, inhibits DENV infection across all four serotypes. TFP was also found to reduce ZIKV infection in several human cell lines. Fluorescent imaging indicated that TFP interferes with both viral entry and uncoating processes, effectively reducing DENV infection [67]. Fluoxetine, a selective serotonin reuptake inhibitor commonly prescribed for manic depression, has demonstrated significant inhibition of DENV RNA replication in both human and murine cell lines when coadministered with the virus. This mechanism leads to reduced virion production and viremia during the early stages of infection. Remarkably, fluoxetine selectively inhibits DENV replication, with no activity against JEV or WNV [68].

Additionally, studies revealed that imipramine, a tricyclic antidepressant (TCA), not only reduced DENV virion production but also inhibited the cleavage of caspase-1 and interleukin-1β (IL-1β) induced by DENV. These effects collectively contributed to the prevention of DENV-induced pyroptosis in dermal fibroblast cells [69].

Collectively, these findings suggest that nervous system-acting agents may serve as potential candidates for host-directed antiviral therapy.

## 5. Nervous System-Acting Compounds With Anti-DENV Activity Potential

Several nervous system-acting compounds have been reported to exhibit antiviral properties, particularly against flaviviruses. To better elucidate their potential mechanisms of action against DENV. We classified these compounds based on their host-targeted antiviral strategies. Specifically, we divided them into two major categories: (1) compounds targeting viral binding and entry and (2) compounds targeting the autophagy pathway, which plays diverse roles during viral replication and maturation. This classification facilitates a clearer understanding of how these drugs may interfere with distinct stages of the DENV life cycle through host-directed mechanisms.

### 5.1. Targeting Viral Binding and Entry

The initial step of DENV infection involves receptor-mediated binding to the host cell surface. Consequently, targeting DENV-associated receptors to block viral entry has emerged as a promising strategy for antiviral intervention. Given its CNS activity and receptor interactions, haloperidol (HALO) is a butyrophenone-class antipsychotic synthesized in the late 1950s and widely used for the treatment of schizophrenia. HALO has demonstrated antiviral activity against JEV through inhibition of the D2R-mediated signaling pathway. Human BE(2)-C neuroblastoma cells and LUHMES dopaminergic neurons showed that HALO treatment significantly reduced the expression of viral NS3 protein and suppressed the production of mature virions. Furthermore, JEV-infected AG129 mice revealed that intravenous administration of HALO not only delayed disease onset but also improved survival outcomes. These findings support the therapeutic potential of HALO in limiting neurotropic flavivirus infections by targeting host dopaminergic signaling [66].

Meanwhile, another study reported that fluphenazine is a phenothiazine antipsychotic structurally related to CPZ and PCZ. It significantly reduced St. Louis encephalitis virus (SLEV) viral load in both Vero cells and human brain endothelial cells. This effect was confirmed by dose–response assays and plaque reduction tests. The authors suggested that its antiviral mechanism may involve inhibition of clathrin-mediated endocytosis [70]. A previous study showed that fluphenazine inhibits clathrin-mediated endocytosis and prevents HCV entry. The authors found that treatment of Huh-7.5 cells with fluphenazine significantly reduced viral entry without affecting cell viability, suggesting that the compound interferes with an early step of the viral life cycle [71]. In a cell-based antiviral assay using Huh7.5 cells, the authors evaluated the antiviral activity of various phenothiazine derivatives against HCV. Both fluphenazine and thioridazine exhibited anti-HCV effects. Although the role of thioridazine was not further explored, the authors reported that fluphenazine inhibits viral entry by disrupting cholesterol-rich regions of the host cell membrane [72].

### 5.2. Targeting the Autophagy Pathway

Upon infection, DENV enters host cells via receptor-mediated endocytosis. Within the endosome, pattern recognition receptors (PRRs) detect viral components and activate signaling pathways involving TRIF and MYD88. This signaling induces autophagy and promotes the formation of double-membrane autophagosomes. Normally, virus-containing autophagosomes fuse with lysosomes to facilitate viral degradation. However, DENV has been shown to subvert this process by utilizing autophagosomes as platforms for viral RNA replication [73]. As a result, targeting autophagy induction or promoting autophagosome degradation has emerged as a potential therapeutic strategy against DENV infection.

The TCA amitriptyline has been shown to enhance autophagosome formation without facilitating the autophagosome–lysosome fusion in A549 lung cancer cells, highlighting its potential to modulate autophagy at a specific stage of the pathway. [74]. Moreover, amitriptyline has demonstrated antiviral activity against JEV by inhibiting viral entry in pretreated Huh-7 cells through suppression of acid sphingomyelinase (ASM), an enzyme that hydrolyzes sphingomyelin into ceramide. Inhibition of ASM reduces ceramide levels at the plasma membrane, thereby interfering with membrane remodeling, which prevents viral entry and replicated [75].

Methotrimeprazine (MTP), also known as levomepromazine, is an antipsychotic drug primarily used to treat schizophrenia. MTP treatment has been shown to reduce JEV infection in cell-based assays and to attenuate JEV-induced mortality and disease severity in murine models. Notably, MTP and TFP did not block JEV entry into host cells. Instead, both compounds were associated with the upregulation of autophagy-related genes, including Atg12, Atg16l1, LC3A, and LC3B. These findings suggest that MTP and TFP may inhibit JEV replication by enhancing autophagy, thereby suppressing viral protein synthesis and replication complex formation [76].

Clomipramine is a TCA that can disrupt autophagic flux by blocking autophagosome–lysosome fusion. This mechanism prevents the formation of functional autolysosomes, which are essential for efficient viral replication [77]. Recently, clomipramine has been suggested as a potential antiviral agent against ZIKV [78]. In addition to its possible antiviral effects, clomipramine treatment has been reported to reduce the expression of proinflammatory cytokines such as IL-6, IL-1β, and TNF-α [79], suggesting a potential dual role in both antiviral defense and immunomodulation.

Paroxetine is a selective serotonin reuptake inhibitor. It has been shown to disrupt autophagic flux. This occurs through impairment of lysosomal acidification and inhibition of lysosomal maturation [80]. A machine learning-based screening study identified paroxetine as a potential antiviral agent against YFV. Experimental validation confirmed that paroxetine reduced virion production in YFV-infected Huh-7 cells. However, its antiviral efficacy was lower compared to other candidate compounds identified in the same study [81].

**Figure 1:**
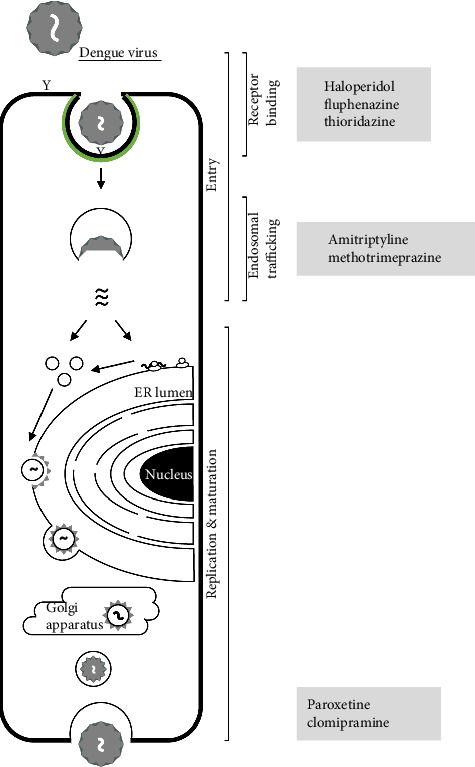
Schematic representation of potential nervous system-acting compounds targeting dengue virus infection at various stages of the viral life cycle.

Collectively, these findings summarized in Table 2 emphasize the potential anti-DENV effects of repurposed nervous system-acting compounds, as also summarized in Figure 1. Further investigation is necessary to clarify their precise mechanisms of action and therapeutic applicability in DENV infection.

## 6. Discussion and Conclusion

Neurological complications associated with DENV infection are rare but can be life-threatening. Given the lack of specific antiviral treatments, drug repurposing presents a promising strategy for identifying novel therapeutic interventions. Antipsychotic medications have emerged as potential candidates for mitigating DENV infection due to their mechanisms of action and ability to penetrate the BBB. In this review, we summarize the potential mechanisms of neuron-targeting medications that may exert antiviral effects against DENV infection. Literature has demonstrated that pharmacological agents targeting D2R, such as CPZ and PCZ, can significantly reduce DENV-induced infection in neuronal and microglial cells both in vitro and in vivo. Importantly, D2R is among the most abundantly expressed dopamine receptors in the CNS, with high expression in neurons and lower but inducible in glial cells under neuroinflammatory conditions. These findings suggest that D2R may serve as a therapeutic target in the management of DENV-induced neurological disease.

On the other hand, typical antipsychotics such as thiothixene and pimozide share a common mechanism of action as dopamine D2 receptor (D2R) antagonists. Recent studies have shown that both compounds exhibit antiviral activity against viruses such as coronaviruses and chikungunya virus [82, 83]. Notably, these antiviral effects appear to be independent of D2R antagonism. However, other D2R antagonists such as HALO, thioridazine, and fluphenazine have shown inhibitory effects against JEV. The antiviral potential of thiothixene and pimozide against flaviviruses has not yet been explored. Further research is needed to evaluate their efficacy against flavivirus infections.

Despite the potential of D2R antagonists as anti-DENV agents, particularly in preventing microglial and neuronal infection, their effects on astrocytes during DENV infection remain unclear. Existing literature suggests that D2R plays a dual role in astrocyte physiology. Under homeostatic conditions, D2R signaling through the αB-crystallin (Cryab) pathway suppresses proinflammatory cytokine secretion and helps maintain immune balance. In contrast, pharmacological blockades of D2R may lead to astrocyte reactivation and enhanced inflammatory responses facilitate neuronal damage [84]. These findings imply that D2R inhibition may act as a double-edged sword, especially in the context of astrocyte-mediated neuroinflammation. Moreover, the role of astrocytes in DENV infection remains controversial. Further studies are needed to clarify the cellular targets of D2R-based therapies and to determine the optimal timing of intervention to maximize antiviral efficacy while minimizing potential neuroinflammatory risks.

However, current evidence regarding the neuropathological mechanisms of DENV infection remains limited. Most existing studies are restricted to in vitro models, which may not fully capture the complex interplay among neurons, microglia, and astrocytes in vivo. As a result, the therapeutic implications of D2R antagonists—particularly their potential benefits versus risks in modulating neuroinflammation—are still largely theoretical. Preclinical studies using animal models are urgently needed to assess the impact of these compounds on CNS immune dynamics during DENV infection. Such investigations will be crucial in determining whether D2R-targeting drugs can be safely repurposed for clinical use in the context of dengue-associated neurological disease.

HALO is currently prescribed primarily for the treatment of schizophrenia and acute agitation. It is also frequently administered for sedation in emergency settings and exhibits good CNS penetration. Structurally, HALO is similar to phenothiazine-class compounds such as CPZ, which has previously demonstrated antiviral activity against DENV. Based on these pharmacological characteristics, HALO is hypothesized to possess potential inhibitory effects on DENV neuro infection.

Fluphenazine is mainly used for the long-term treatment of chronic schizophrenia. In vitro studies have shown that fluphenazine exhibits antiviral activity against SLEV and HCV, suggesting its potential as a repurposed antiviral agent. However, its clinical application requires caution due to the risk of extrapyramidal side effects. Similarly, the clinical utility of thioridazine is significantly limited due to its high risk of cardiotoxicity, particularly QT interval prolongation.

Paroxetine is widely prescribed for the treatment of depression, anxiety, and obsessive-compulsive disorder. It has a high clinical usage rate and a generally favorable safety profile compared to typical antidepressants. However, it is associated with side effects such as gastrointestinal disturbances and sexual dysfunction. In rare cases, serious adverse events including serotonin syndrome and cardiac arrhythmias have been reported.

Other neuroactive drugs, such as clomipramine and amitriptyline, are also widely used in clinical practice. While both are effective, they are associated with notable side effects. Common adverse effects include dry mouth, sedation, constipation, weight gain, and dizziness. Cardiovascular complications, such as hypotension and arrhythmias, are also a concern and can lead to treatment discontinuation. Clomipramine, in particular, may lower the seizure threshold, especially at higher doses.

In summary, HALO stands out as the most clinically accessible and widely used candidate with theoretical potential against DENV neuroinfection, given its pharmacological profile and established safety record (Table 3). Fluphenazine also warrants further investigation due to its in vitro antiviral efficacy. Nonetheless, additional research is required to elucidate the underlying antiviral mechanisms of these compounds, as well as to determine whether the effective concentrations observed in experimental models are clinically achievable. Moreover, their therapeutic potential and safety, including dosing limitations, must be thoroughly evaluated in preclinical and clinical settings. Neuroactive drug repurposing represents one strategy among broader efforts, including vaccination and vector control, to combat dengue infection.

## Figures and Tables

**Table 1:** Comparison between the 2009 and 1997 WHO dengue classification systems.

Classification system	Grade/category	Clinical features
WHO 2009 Dengue guidelines	Dengue without warning signs	Headache, muscle pain, joint pain, rash
	Dengue with warning signs	Abdominal pain or tenderness, persistent vomiting, fluid accumulation (ascites, pleural effusion), mucosal bleeding, lethargy or restlessness, hepatomegaly
	Severe dengue	Severe plasma leakage leading to shock or respiratory distress, severe bleeding, or severe organ impairment (liver, brain, heart)

WHO 1997 Dengue guidelines (1-5 grading System)	Grade 1	Mild fever, headache, muscle pain
	Grade 2	Mild bleeding symptoms, no shock or severe organ damage
	Grade 3	Significant thrombocytopenia, plasma leakage, pre-shock phase
	Grade 4	Shock phase (dengue shock syndrome), hypotension, organ dysfunction
	Grade 5	Severe complications (severe bleeding, liver failure, encephalopathy)

*Note:* Data adapted from WHO 1997 and WHO 2009 Dengue guidelines.

**Table 2:** Summarize the possible mechanisms of nervous system-acting compounds for anti-DENV infection.

Drug	Susceptible virus	Effective concentration	Model	Possible antiviral mechanism	References
Haloperidol	JEV	20 μM	BE(2)C cell	Inhibiting D2R-mediated viral entry	[66]

Fluphenazine	SLEV	20 μM	Vero/HBEC-5i	Inhibiting clathrin-mediated endocytosis	[70]
	HCV	0.5 μM	Huh-7.5	Inhibiting clathrin-mediated endocytosis	[71]
	HCV	0.37 ± 0.01 μM (IC_50_)	Huh-7.5	Inhibiting D2R-mediated viral entry	[72]

Thioridazine	HCV	0.78 ± 0.31 μM (IC_50_)	Huh-7.5	Unknown	[72]

Amitriptyline	JEV	20–40 μM	Huh-7.5	Inhibiting viral entry	[75]

Methotrimeprazine	JEV	10 μM	Neuron-2A, BMDM, primary cortical neurons	Suppressing viral RNA replication through the induction of autophagy	[76]

Clomipramine	ZIKV	—	—	Unknown	[78]

Paroxetine	YFV	3.2 μM	Huh-7.5	Unknown	[81]

Abbreviation: BMDM: bone marrow-derived macrophage.

**Table 3:** Summarize the clinical usage and frequency of nervous system-acting compounds.

Drug	Use frequency	Dosage (mg/day)	Primary indications	Toxicities	Major risks	Ref
Haloperidol	^∗∗∗^	1–15	Schizophrenia	EPS, Sedation	Arrhythmia, neuroleptic malignant syndrome	[85, 86]
Amitriptyline	^∗∗∗^	25–150	Depression, neuropathic pain	Sedation, anticholinergic effects	Arrhythmia, orthostatic hypotension	[87]
Paroxetine	^∗∗∗^	20–50	Depression, anxiety	GI discomfort, sexual dysfunction, sedation	Serotonin syndrome, arrhythmia	[87]
Fluphenazine	^∗∗^	1–40	Schizophrenia	EPS, sedation,	Cardiovascular effects	[85, 86]
Clomipramine	^∗∗^	25–250	Depression	Anticholinergic effects, sedation	Arrhythmia, seizure threshold lowering	[87]
Thioridazine	^∗^	50–800	Schizophrenia	EPS, sedation, anticholinergic effects	Cardiotoxicity (QT prolongation), arrhythmia	[85, 86]

*Note:* EPS, extrapyramidal symptoms; GI, gastrointestinal; clinical usage frequency is indicated as follows: ^∗∗∗^ = high, ^∗∗^ = moderate, ^∗^ = low.

## Data Availability

No data are available for this review manuscript.
